# Surgical intensive care unit clinician estimates of the adequacy of communication regarding patient prognosis

**DOI:** 10.1186/cc9346

**Published:** 2010-11-29

**Authors:** Rebecca A Aslakson, Rhonda Wyskiel, Dauryne Shaeffer, Marylou Zyra, Nita Ahuja, Judith E Nelson, Peter J Pronovost

**Affiliations:** 1 Department of Anesthesiology and Critical Care Medicine, The Johns Hopkins School of Medicine, 600 North Wolfe Street, Baltimore, MD, 21287, USA; 2 Department of Surgical Nursing, The Johns Hopkins Hospital, 600 North Wolfe Street, Baltimore, MD, 21287, USA; 3 Department of Surgery and Oncology, The Johns Hopkins University School of Medicine, 600 North Wolfe Street, Baltimore, MD, 21287, USA; 4 Department of Medicine, Division of Pulmonary, Critical Care and Sleep Medicine and Hertzberg Palliative Care Institute, Mount Sinai School of Medicine, One Gustave L. Levy Place, New York, NY, 10029, USA

## Abstract

**Introduction:**

Intensive care unit (ICU) patients and family members repeatedly note accurate and timely communication from health care providers to be crucial to high-quality ICU care. Practice guidelines recommend improving communication. However, few data, particularly in surgical ICUs, exist on health care provider opinions regarding whether communication is effective.

**Methods:**

To evaluate ICU clinician perceptions regarding adequacy of communication regarding prognosis, we developed a survey and administered it to a cross section of surgical ICU nurses, surgical ICU physicians, nurse practitioners (NPs), and surgeons.

**Results:**

Surgeons had a high satisfaction with communication regarding prognosis for themselves (90%), ICU nurses (85%), and ICU physicians and NPs (85%). ICU nurses noted high satisfaction with personal (82%) and ICU physician and NP (71%) communication, but low (2%) satisfaction with that provided by surgeons. ICU physicians and NPs noted high satisfaction with personal (74%) and ICU nurse (88%) communication, but lower (23%) satisfaction with that provided by surgeons. ICU nurses were the most likely (75%) to report speaking to patients and patient families regarding prognosis, followed by surgeons (40%), and then ICU physicians and NPs (33%). Surgeons noted many opportunities to speak to ICU nurses and ICU physicians and NPs about patient prognosis and noted that comments were often valued. ICU physicians and NPs and ICU nurses noted many opportunities to speak to each other but fewer opportunities to communicate with surgeons. ICU physicians and NPs thought that their comments were valued by ICU nurses but less valued by surgeons. ICU nurses thought that their comments were less valued by ICU physicians and NPs and surgeons.

**Conclusions:**

ICU nurses, surgeons, and ICU intensivists and NPs varied widely in their satisfaction with communication relating to prognosis. Clinician groups also varied in whether they thought that they had opportunities to communicate prognosis and whether their concerns were valued by other provider groups. These results hint at the nuanced and complicated relationships present in surgical ICUs. Further validation studies and further evaluations of patient and family member perspectives are needed.

## Introduction

Intensive care unit (ICU) patients and their family members repeatedly identify communication as essential to high-quality ICU care [1,2]. They emphasize the importance of "timely, clear, and compassionate communication by clinicians" [3]. Most want to know their physicians' estimates of prognosis, even if the prognosis is uncertain [4-6]. Patients and families benefit from proactive communication because it decreases the use of unwanted and ineffective treatments in the ICU [7,8], reduces ICU length of stay [9], and promotes earlier consensus around goals of care [10]. Consequently, professional societies and practice guidelines recommend that ICU clinicians communicate proactively with patients and patient families [11-15].

As in other settings, optimal communication in the ICU includes discussion of the disease, prognosis, goals of care, treatment options, and patient preferences. Proactive ICU communication should also address preferences regarding resuscitation. When burdens of intensive care treatment outweigh benefit, discussion of limitation of this treatment is also appropriate. Evidence suggests that clinicians working in surgical ICUs find this type of communication to be particularly challenging. Surgical textbooks contain scant content related to communication of distressing news or goal setting [16]. The "rescue culture" that dominates many surgical ICUs may also further impede such discussions [17-20]. Moreover, as these discussions can reveal varying goals of care between health care providers, they often cause moral distress and conflict [21,22].

Data from our own institution suggest that patients receiving surgical ICU care for more than 7 days have a high (41%) rate of in-hospital mortality [23]. Thus, the specific aim of this article is to investigate the views and experiences of clinicians working in the surgical ICU regarding the adequacy and efficacy of communication regarding prognosis for surgical ICU patients.

## Materials and methods

In September and October of 2009, we completed a cross-sectional, computer-based survey to assess health care-provider opinions concerning communication regarding prognosis. We surveyed physicians, nurses, nurse practitioners (NPs), and house staff at the Johns Hopkins Hospital who admit to or practice in one of three surgical ICUs: a 15-bed cardiac surgical ICU that predominantly admits patients after cardiac surgery; a 13-bed surgical ICU and intermediate care unit (IMC) that predominantly admits patients after trauma, transplant, and vascular surgeries; and a 16-bed general surgical ICU and IMC that predominantly admits patients after thoracic, general abdominal, plastic, gynecologic, and ear/nose/throat surgeries. Each surgical ICU operates under a "semi-open" plan; patients are admitted by the primary surgeon with his or her corresponding house-staff team, but with a required ICU team consultation. Thus, decisions are made jointly between the primary surgical and the ICU team. Attending surgeons are board certified or board eligible in surgery. The ICU attending team is interdisciplinary and comprises predominantly physicians with primary board certification in either surgery or anesthesia (a few have primary board certification in medicine or emergency medicine); the majority also concurrently have board certification or are board eligible in critical care. The ICU team comprises either house staff or nurse practitioners, with nurse practitioners having responsibilities similar to those of senior house staff.

Participation in the survey was voluntary. The Johns Hopkins University School of Medicine Institutional Review Board (IRB) approved this research and waived the need for informed consent.

### Survey development

**Figure 1:**
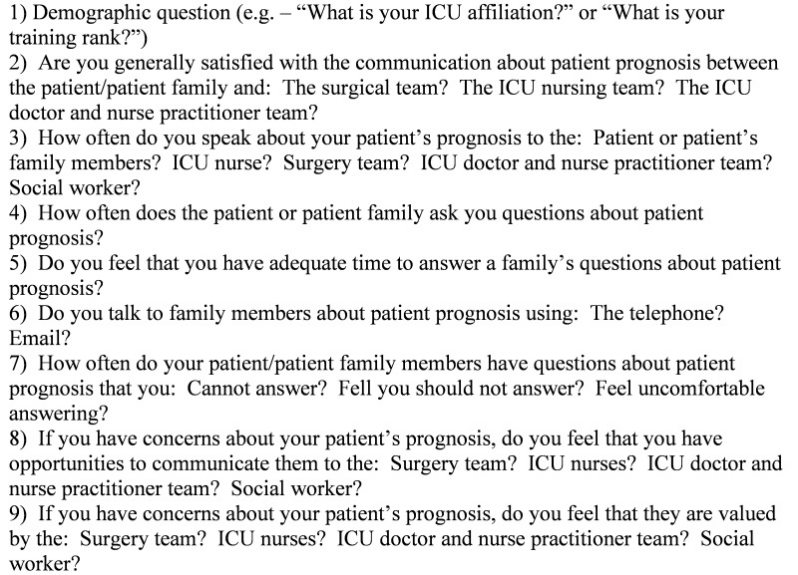
**Survey instrument**.

To identify domains for the survey questionnaire, we conducted a literature review and convened focus groups of physicians (surgeons and intensivists) and nurses (ICU staff nurses and NPs) at our academic medical center. The literature review was conducted in PubMed by exploding the terms "communication" and "intensive care unit" and reviewing relevant hits as well as by hand-searching personal files. Focus groups were small (two to three individuals), voluntary, and convened with a study investigator (RA) as moderator; comprising nurses, ICU attendings, ICU nurse practitioners, and surgeons who expressed an interest in communication in the ICU, groups were informally asked to list important factors in assessing whether communication between health care providers, and between health care providers and patients, is adequate. The research team reduced and refined the list to include three domains: satisfaction with communication, frequency of communication, and valuation of communication. Within these domains, specific questions called for categoric responses, by using either a nominal ("yes/no") or adjectival Likert scale ("multiple times/day, daily, sometimes, rarely, never" or "always, daily, sometimes, rarely, never"). We pilot tested this questionnaire with a sample of six clinicians, including ICU and surgery physicians and ICU nurses. Based on feedback, the final survey was revised to contain nine questions (Figure 1). At the beginning of our survey, the following definition was given: "For this survey, 'prognosis' is specifically defined as how a patient's illness and overall health is likely to evolve during this hospitalization or over the next few days to months."

### Study sample and method of survey administration

The study sample included full-time ICU nurses, ICU intensivists, or NPs who work in any of the previously described three ICUs; it also included surgeons who admit to any of these three ICUs. All care providers were contacted via an e-mail containing a link to the survey Web page. Potential survey participants were identified by rosters listing full-time employed surgical ICU nurses, intensive care unit attendings or fellows, intensive care unit advanced nurse practitioners, surgeons, surgery house staff, and anesthesia house staff. The survey was facilitated by the Web-based service, SurveyMonkey [24].

### Survey statistics and sample size

Surveys were pooled by group--surgeons, ICU nurses, and ICU physicians and NPs--with the outcomes listed as proportions. As the NP role is similar to that of senior ICU house staff, NP responses were listed with the ICU physician group, as opposed to the ICU nurse group. Descriptive statistics were used. Stata software (version 10.1; College Station, TX) was used for all analyses.

## Results

We received 103 responses from a pool of 258 individuals (overall response rate, 40%). Subclassification by group included a total of 40 responses from surgical ICU nurses (47% response rate), 39 responses from ICU physicians (52%), four responses from nurse practitioners (NPs; 50% response rate), and 20 responses from surgeons (22% response rate). Further demographics of survey respondents are depicted in Table 1.

**Table 1:** Demographics of survey respondents

Surgeons (*n *= 20)		ICU physicians and NPs (*n *= 41)		ICU staff nurses (*n *= 40)	
		
Rank	Number (%)	Rank	Number (%)	SICU affiliation	Number (%)
Attending surgeon	6 (30)	Attending intensivist	17 (41.5)	Cardiac SICU	15 (37.5)
Surgeon and ICU attending	1 (5)	Nurse practitioner	4 (9.8)	Trauma, transplant, vascular SICU	8 (20)
Fellow	2 (10)	Fellow	1 (2.4)	General SICU	17 (42.5)
Chief of resident service or senior resident	7 (35)	Resident	19 (46.3)		
Junior resident or intern	4 (20)				

ICU, intensive care unit; NP, nurse practitioners; SICU, surgical intensive care unit.

### Satisfaction with communication regarding prognosis

Among respondents who were surgeons, the vast majority were satisfied with their own communication and the communication by ICU physicians, NPs, and ICU staff nurses (Table 2). Three fourths of ICU physicians and NPs were satisfied with their own communication about prognosis; nearly nine of ten were satisfied with that of ICU nurses; and just less than one fourth were satisfied with surgeon communication regarding prognosis. Of ICU nurses, many were satisfied with their own communication and ICU physician and NP communication, and few (3%) were satisfied with surgeon communication regarding prognosis.

**Table 2:** Health care provider satisfaction with communication regarding prognosis

	Person Reviewed		
	
Reviewer	Surgeon	ICU MD/NP	ICU RN
Surgeon	90%	85%	85%
ICU MD/NP	23%	74%	88%
ICU RN	3%	71%	82%

ICU, intensive care unit; MD, physician; NP, nurse practitioner; RN, nurse.

### Perceptions of frequency of discussions with families

Three fourths of ICU nurses report speaking to patients and patient families daily or multiple times per day regarding prognosis. Forty percent of surgeons and one third of ICU physicians and NPs report speaking to patients and patient families daily or multiple times per day regarding prognosis.

### Perceptions of frequency of discussions with other health care providers

The different types of health care providers varied in how often they reported speaking to each other regarding a patient's prognosis (Table 3). Just less than half of ICU nurses noted that they and surgeons speak to each other about a patient's prognosis daily or multiple times per day, whereas 60% of surgeons noted the same conversations occurring. Among ICU physicians and NPs and surgeons, half of ICU physicians and NPs noted that they speak to the surgeon about a patient's prognosis on a daily or multiple times per day basis, whereas a higher percentage of surgeons noted the same conversations occurring. ICU physicians and NPs and ICU nurses noted similar occurrences of speaking to each other daily or multiple times per day regarding a patient's prognosis.

**Table 3:** Frequency of discussions concerning prognosisa

	Recipient		
	
Communicator	Surgeon	ICU MD/NP	ICU RN
Surgeon	-	80	60
ICU MD/NP	53	-	81
ICU RN	43	85	-

^**a**^**Percentage noting "daily or multiple times a day" discussions**. ICU, intensive care unit; MD, physician; NP, nurse practitioner; RN, nurse.

### Opportunities for and valuation of discussions

Ninety percent of surgeons responded that they had multiple opportunities per day to speak with both ICU physicians and NPs and ICU nurses regarding patient prognosis (Table 4). The majority of surgeons also reported that their concerns regarding prognosis were valued by both ICU physicians and NPs and ICU nurses. ICU nurses noted many opportunities to communicate with ICU physicians and NPs, although many thought that their comments were moderately valued by ICU physicians and NPs. ICU nurses noted fewer opportunities to communicate to surgeons and, again, that their concerns were not as valued by surgeons. ICU physicians and NPs noted many opportunities to communicate with ICU nurses and that their concerns were valued by the nurses. However, ICU physicians and NPs had fewer opportunities to communicate with surgeons, and they thought that their comments were less valued.

**Table 4:** Opportunities between health providers to communicate regarding patient prognosis and whether that communication is valued

	Recipient					
	
Communicator	Surgeon		ICU MD/NP		ICU RN	
			
	Opportunity to communicate	Communication valued	Opportunity to communicate	Communication valued	Opportunity to communicate	Communication valued
Surgeon	-	-	90%	75%	90%	90%
ICU MD/NP	46%	27%	-	-	85%	83%
ICU RN	29%	31%	91%	63$	-	-

The percentages given indicate "always" or "often." ICU, intensive care unit; MD, physician; NP, nurse practitioner; RN, nurse.

## Discussion

ICU nurses, surgeons, and ICU intensivists and nurse practitioners varied widely in their satisfaction with communication relating to prognosis. Moreover, health care provider groups also varied in whether they thought that they had opportunities to communicate prognosis and whether their concerns were valued by the other providers. In total, these results hint at the nuanced and complicated relationships present in ICUs, particularly surgical ICUs, where nurses, surgeons, and ICU physicians and NPs must work together to provide the care for any single patient.

Previous studies quantify and qualify varying physician and nurse perceptions regarding prognosis and end-of-life care [25,26] as well as illustrate that optimal collaboration between physicians and nurses decreases clinician moral distress [27,28], prevents clinician burnout [29], and improves patient outcomes [30,31]. Moreover, multiple factors, including the educational status of the patient's family, the racial background of the physician, physician uncertainty regarding the patient's prognosis, and conflicts between the patient family and the physician, affect whether physicians discuss prognosis and the language used in that discussion [5,6,32,33]. Finally, not all patient families wish to discuss prognosis [34,35] and health care providers should consider using the "ask-tell-ask" protocol to elicit family member preferences regarding discussions of prognosis [36,37].

Among ICU nurses as well as ICU physicians and NPs, general dissatisfaction existed with how surgeons communicate prognosis, the opportunities to communicate with surgeons regarding prognosis, and whether the conversations were valued by the surgeon. Yet, surgeons reported good communication between themselves and nurses and ICU physicians and NPs. A number of potential causes could be contributing to this. First, the surgeons who responded to the survey could be already interested in communication and thus have better communication skills than their colleagues. Furthermore, it is unclear whether the dissatisfaction is from a lack of communication regarding prognosis or whether the content of the discussion was not thought appropriate (perhaps either overly optimistic or overly pessimistic). ICU physicians, NPs, and nurses may not be aware of discussions surgeons have with patients. Finally, surgeon perceptions of the need for discussing prognosis and comfort with such discussions may also differ from that of ICU nurses, NPs, and physicians.

The advent of ICU daily-goal sheets [38] ensures that communication between the ICU physician and NP team and the ICU nursing team is both timely and follows a protocol. However, goal sheets rarely exist to prompt similar detailed conversations between surgeons and ICU nurses and/or surgeons and ICU physicians and NPs; the miscommunication or lack of communication between surgeons and ICU providers may be a consequence of this lack of a daily goal sheet.

Further cultural reasons may underlie these discrepancies. For example, the concept of "prognosis" may differ between health care provider groups. In her book, *Life and Death in Intensive Care *[20], Joan Cassell notes that, particularly for patients in surgical ICUs, surgeons, ICU physicians, and nurses often have different "moral economies." In Dr. Cassell's description, surgeons note the enemy to be death, and emphasis is subsequently placed on caring for the physiologic patient with a goal of averting death at all costs. In contrast, ICU physicians and nurses envision the greatest enemy to be suffering, and emphasis is toward averting suffering, particularly if a patient is dying and the suffering is seen as "needless." These different moral economies may derive from the increased time that nurses and ICU teams, as compared with surgeons, spend with suffering ICU patients.

Although Cassell's book and similar articles by Buchman *et al. *[18] and Penkoske and Buchman [19] address end-of-life care, similar principles may also hold true for discussions regarding prognosis. When asked about "communication regarding prognosis," surgeons could be interpreting "prognosis" as being whether the patient can be kept alive for the next day, or until discharge from the surgical ICU or hospital. In contrast, ICU nurses and ICU physicians and NPs could be interpreting "prognosis" to mean whether the patient is likely to have a long hospitalization, a complicated post-hospitalization course of rehabilitation, or the ability for the patient to have a quality of life that is consistent with his or her values and beliefs. Despite having defined "prognosis" in the survey, the term could have been differently interpreted.

Cultural environments--such as differences between surgical, nursing, and ICU physician/NP cultures--can also simplify, or complicate, discussions regarding prognosis. Besides an emphasis on preventing death, aspects of surgical culture that could complicate the communication of prognosis include: the "rescue credo" [17]--the need to "save" or "rescue" a patient from dying; that surgeons often associate patient death with personal failure and shame [17,20] and professional "shame" if a patient does not receive all possible interventions [18].

Less is found in the literature concerning the culture of ICU physicians and NPs, particularly in surgical ICUs. ICU physicians, some of whom are surgeons, and NPs, can have varying opinions on whether the goal of surgical ICU care is to avert death, to avert suffering, or some balance of the two. Moreover, individual opinions within caregiver groups likely vary widely (for example, not all surgeons or nurses have the same views regarding prognosis), and little is known regarding the relative size of within-group versus between-group variation. Moreover, ICU physicians often rotate weekly, leaving little time to bond with families and to discuss prognosis [20]. Nevertheless, little empiric evidence exists that this ICU-physician schedule poses barriers to discussing prognosis.

The ICU nurse has a pivotal role for ICU patients and their families. From a practical standpoint, nurses spend the most time with ICU patients and family members [39], and thus are a valuable resource for identifying what the patient and family understand and whether communication with them is needed or effective. Moreover, nurses often informally provide families with information about prognosis and act as the medical "translator" [20] for families, as well as the "power broker" between health care providers, particularly if surgeon and ICU physician and NP teams disagree. Despite this, ICU nurses are often excluded from formal discussions regarding prognosis, limiting their ability to inform these discussions. Such situations can contribute to the all-too-common phenomenon of a patient or patient family hearing contrasting prognoses from varying health care providers.

Multiple limitations to this study exist. First, our sample size was small, and we studied staff from surgical ICUs in one academic medical center; our findings may not be representative of the views of clinicians working in other settings. Second, given the response rates, we cannot exclude the possibility of significant response and nonresponse bias in the survey results. Third, although we provided a working definition of prognosis for purposes of the survey, clinicians may have understood the term differently based on their prior experience. Fourth, our survey instrument was not previously validated; however, to our knowledge, no existing validated tools address these specific issues, and our methods for survey development supported the validity of the instrument we used. Finally, the study measures perceptions and estimations of communication, which is potentially unreliable. However, studies measuring actual quality and content of this communication are cumbersome, and, as perceptions and knowledge drive actions, we consider it worthwhile to assess those perceptions.

The results of our study highlight potential targets for ICU-performance improvement initiatives. Daily goal sheets for surgeons and ICU teams (nurses and/or ICU physicians and NPs) could direct the content of conversations as well as facilitate more frequent opportunities for discussions. Moreover, routine multidisciplinary family meetings for ICU patients and patient families could further mitigate deficiencies highlighted by the surveys. Finally, as palliative care teams become more prominent in the ICU, or as surgeons, ICU physicians and NPs, and ICU nurses become more skilled in palliative care, such discussions regarding prognosis may also become less problematic.

Although optimal communication in the ICU is difficult, it is especially difficult in surgical ICUs where patients are often cared for by two sets of physicians. This study highlights the need for further additional research in surgical ICU communication, particularly exploring patient and patient family opinions, and for focused efforts to improve communication.

## Conclusions

ICU nurses, surgeons, and ICU intensivists and NPs varied widely in their satisfaction with communication relating to prognosis. These same clinician groups also varied in whether they thought that they had opportunities to communicate prognosis and whether their concerns were valued by other clinicians. These variations could result from practical circumstances (some groups may be unaware of discussions that do occur); system failures (tools that facilitate discussions, such as daily goal sheets, may not exist); or cultural differences (different groups have varying expectations about when communication should occur and what should be the content of that communication). Further research, particularly into the communication expected and desired by surgical ICU patients and their families, is needed.

## Key messages

• Optimal care for surgical ICU patients requires clear communication between ICU nurses, surgeons, and ICU physicians and mid-level providers.

• Not all health care provider teams in surgical ICUs are satisfied with how other teams communicate prognosis to patients.

• ICU nurses are the most likely to report speaking at least daily to patients and patient families about prognosis.

• Some health care provider teams, particularly ICU nurses, report often not having opportunities to communicate about patient prognosis, and that when communication does occur, their input is less valued by other providers.

• Varying levels of health care provider satisfaction in communication regarding prognosis may be a result of practical barriers, such as a lack of daily goal sheets spurring communication between surgeon and ICU teams, as well as cultural barriers, such as whether a "bad prognosis" specifically refers to imminent patient death or may instead refer to patient suffering, such as the inability for a patient to recover a quality of life that is consistent with his or her values and beliefs.

## Abbreviations

ICU: intensive care unit; IMC: intermediate care unit; MD: physician; NP: nurse practitioner; RN: registered nurse; SICU: surgical intensive care unit.

## Competing interests

The authors declare that they have no competing interests.

## Authors' contributions

RA conceived and designed the studies, wrote and distributed the surveys, analyzed the data, and drafted the manuscript. RW distributed surveys, analyzed data, and was influential in drafting the manuscript. DS distributed surveys. MZ distributed surveys and analyzed data. NA distributed surveys, provided idea content for the discussion section of the manuscript, and was influential in the drafting of the manuscript. JN aided in conception of the study and provided idea content for the discussion section of the manuscript. PP aided in conceiving and designing the study, analyzed the data, and provided idea content for the discussion section of the manuscript. All authors read and approved the final manuscript.
